# Transcutaneous Non-Invasive Vagus Nerve Stimulation: Changing the Paradigm for Stroke and Atrial Fibrillation Therapies?

**DOI:** 10.3390/biom14121511

**Published:** 2024-11-26

**Authors:** Carola Y. Förster

**Affiliations:** Department of Anaesthesiology, Intensive Care, Emergency and Pain Medicine, Cerebrovascular Sciences and Neuromodulation, Würzburg University, 97080 Würzburg, Germany; foerster_c@ukw.de; Tel.: +49-931-201-30065

**Keywords:** stroke, neuroplasticity, atrial fibrillation, cardiovascular diseases (CVD), inflammation, vagus nerve, taVNS

## Abstract

A new therapeutic approach, known as neuromodulation therapy—which encompasses a variety of interventional techniques meant to alter the nervous system in order to achieve therapeutic effects—has emerged in recent years as a result of advancements in neuroscience. Currently used methods for neuromodulation include direct and indirect approaches, as well as invasive and non-invasive interventions. For instance, the two primary methods of stimulating the vagus nerve (VN) are invasive VN stimulation (iVNS) and transcutaneous VN stimulation (tVNS). Since the latter is non-invasive, basic, clinical, and translational studies have focused on transcutaneous auricular VN stimulation (taVNS), the primary tVNS therapy, because of its advantages over iVNS, including ease of use, greater accessibility, and a lower side effect profile. taVNS is currently used as a novel neuromodulatory application to treat cardiovascular, mental, and autoimmune diseases. Future applications of this non-invasive neuromodulation technology to conditions like atrial fibrillation (AF) or ischemic stroke are highly likely due to its advancement.

## 1. Electrical Neuromodulation as Biophysical Medicine—Introduction and Historical Review

Modern medicine has achieved great success in areas such as emergency medicine, surgical interventions, prophylaxis, imaging diagnostics, etc. Nevertheless, progress seems to be stagnating in other areas, including chronic diseases. There are even new diseases, such as chemical intolerance, chronic fatigue syndrome, high blood pressure, and psycho-organic syndrome. There are still no clear causes of these diseases and therefore hardly any effective therapies. This also includes the increasing number of allergies, chronic inflammation, and many types of cancer. There are reports of successful healings from various areas of biophysical medicine (electrotherapy as complementary medicine) [1].

**Figure 1:**
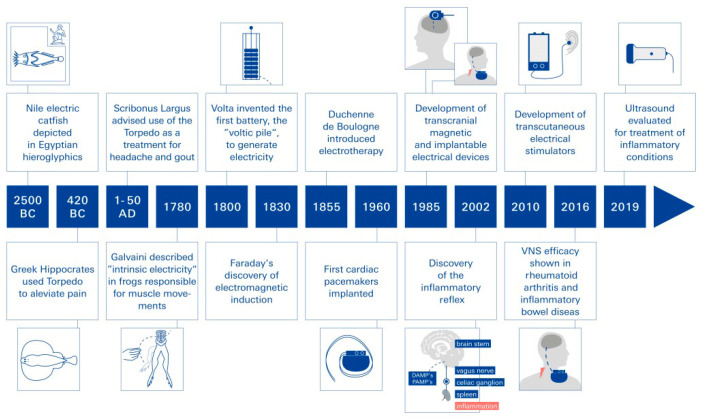
A brief history of bioelectronic medicine. Since antiquity, a number of invasive and non-invasive electrical stimulators have been developed to treat specific diseases as a result of sequential discoveries.

Historically, the treatment of inflammation and pain using non-invasive electrical neuromodulation can be traced back about 5000 years, as Egyptian grave reliefs (ca. 2500 BCE) prominently depict the Nile catfish (Malopturus) and refer to its use in painful diseases (Figure 1). The effects of “natural electricity” from contact with the electric ray (torpedo fish) were described as narcotic by Hippocrates and Aristotle. The first person known to have been cured of an inflammatory disease by electricity was Anteros, a court official of the Emperor Tiberius (42 BC): while walking along a seashore, he accidentally stepped on a torpedo fish and received a strong electric shock (torpedo voltages range up to 225 volts). After the narcotic effect of the shock wore off, Anteros realized he was freed from his gout, a type of arthritis. In reference to this incident, Scribonius Largus, the court physician of the Roman Emperor Claudius, suggested using the torpedo fish’s electric shocks as a therapy for arthritis, making the electric fish the first non-invasive neuromodulation device used by man to treat inflammatory diseases [2].

Neuromodulation as therapy in modern times. Bioelectronic devices that make use of electrical neuromodulation have been used for decades to treat various diseases [1]. Based on the sequential discoveries of bioelectricity by Galvani (1780), the battery by Volta (1799), and electromagnetic induction by Faraday (1831), a variety of invasive and non-invasive electrical stimulators have been developed to treat specific diseases (Figure 1). For example, deep brain stimulation improves the quality of life of patients with depression or Parkinson’s disease; sacral nerve stimulation helps people with bowel and bladder problems; pacemakers and defibrillators have revolutionized the treatment of patients with cardiac arrhythmias; and vagus nerve stimulation (VNS) has been used for more than 30 years to treat drug-resistant seizures [2] (Figure 1). Other successful forms of neuromodulation include transcranial magnetic stimulation and ultrasound technology. In addition, the use of tumor treating fields (TTFs) has been established in cancer therapy for some time now. These fields appear to interfere with the spindle apparatus formation and thus the proliferation of cancer cells [2,3]. Specifically, the permeability of the blood–brain barrier (BBB) was altered by the tumor-treating fields (TTField) application, which implies that this could be a viable therapeutic approach for glioblastoma multiforme (GBM) treatment by facilitating drug delivery across the BBB [3].

## 2. Vagus Nerve Stimulation (VNS)

Since the 20th century, VNS and specifically tVNS therapies have gained popularity and established themselves as significant neuromodulation therapies in the field of neuroscience thanks to advancements in pertinent basic research and conceptual framework revision [4]. The VN is the longest cranial nerve in the human body; it extends from the brainstem into the abdomen. The VN is extensively dispersed and plays a crucial role in regulating numerous physiological functions. As the main conduit of the parasympathetic branch, the VN affects several organs. By sending signals from the brain to these organs, the VN helps regulate a number of body processes, such as heart rate variability, gastrointestinal (GI) motility, and pancreatic function [5,6,7,8]. Increasing the scientific understanding of the VN and insights into its anatomy and conduction have led to the development of robust treatment approaches [9]. VNS techniques come in both invasive and non-invasive varieties. Invasive VNS is currently approved by the Food and Drug Administration (FDA) in the US for treating pharmacoresistant depression and chronic epilepsy. Clinical trials have shown that a number of VNS treatment approaches have notable antidepressant and seizure-inhibitory effects [10,11]. Recently, the non-invasive taVNS has also been approved by the FDA for the treatment of a number of clinical disorders, including drug-resistant migraines, treatment-resistant depression, and tinnitus [12,13,14,15,16]. taVNS uses the cutaneous distribution of afferent vagus nerve fibers in the auricle (tragus, cymba concha), the auricular branch of the VN (ABVN), and the cervical branch of the VN (CBVN), respectively [17,18]. Understanding the operation of the autonomic nervous system (ANS), and specifically the VN, is crucial to comprehending the fundamentals of tVNS [18]. Both sympathetic and parasympathetic divisions make up the ANS, with the VN constituting the majority of the parasympathetic nervous system.

As of yet, however, taVNS has not been sufficiently investigated in terms of its application and mechanism of action for it to be used clinically on a large scale anytime soon. Furthermore, the taVNS stimulation parameters differ significantly between studies. More coherence in neurostimulation research and its neuroanatomical basis is required, despite the fact that the number of research papers on this subject is increasing. In order to help taVNS become a recognized clinical treatment option that is applied more widely in future clinical practice, this editorial comment aims to highlight pertinent research and innovative clinical treatment options. We will make reference to earlier *Biomolecules* articles that partially describe these options in this insight article’s concluding section. This paper will be particularly focused on the study of the inflammation and neuroplasticity processes associated with stroke, as well as the application of these concepts to cardiovascular diseases. Particularly, atrial fibrillation (AF) [19] will be addressed in this perspective, as ischemic stroke resulting from AF is highly prevalent, presenting with severe neurologic symptoms and carrying a high risk of recurrence.

## 3. taVNS for Stroke Treatment

One of the main causes of disability is ischemic stroke, and there are few treatment approaches that support functional recovery following a stroke. Novel therapies that enhance neurological recovery following a stroke are therefore desperately needed.

It has been demonstrated that in chronic stroke patients, iVNS combined with rehabilitation improves upper limb motor function [20]. But since iVNS necessitates surgery, it might not be appropriate for every stroke patient. Similar vagal nerve projections in the central nervous system have been demonstrated to be activated by non-invasive tVNS through auricular VNS in the ear (taVNS) and cervical VN stimulation in the neck (tcVNS). In animal models of hyperacute stroke, taVNS has demonstrated encouraging results as a means of reducing infarct size and stroke-associated neuroinflammation [21]. In chronic stroke patients, for example, taVNS combined with limb movements appears to improve neurological recovery. In sum, the available data support taVNS in human clinical stroke trials and preclinical models. We here highlight the molecular mechanisms underlying taVNS’s beneficial effects, and we point out the main research directions aimed at integrating taVNS into acute and chronic stroke clinical care [22,23]: taVNS administered in acute middle cerebral artery occlusion reduces infarct size through anti-inflammatory effects, decreased excitotoxicity, and enhanced blood–brain barrier (BBB) integrity, according to a number of pre-clinical studies on the effects of taVNS over time [24,25]. In particular, rats treated with taVNS showed noticeably less serum IgG leakage in the ischemic hemisphere as detected by immunohistochemistry. Additionally, taVNS decreased the expression of matrix metalloproteinases-2/-9 in reactive astrocytes surrounding the compromised vessels in the ischemic hemispheres and prevented vascular tight junction proteins from being disrupted in microvessels [24,25].

## 4. taVNS Treatment Helps to Prevent Post-Stroke Impairments by Promoting Neurological Plasticity

After a stroke, many patients suffer from limitations. These may include paralysis and visual or speech disorders. However, those affected can regain their lost abilities with the help of appropriate therapy. A stroke causes a lack of blood flow to certain regions of the brain due to a vascular blockage or a brain hemorrhage. This means that the nerve cells do not receive an adequate supply of oxygen and nutrients and die. The bodily functions that are controlled by this area of the brain lose their purpose [26]. However, new connections are formed as learning processes enable the brain to reorganize itself.

It is now known that the adult brain can also undergo significant changes. The plasticity of the brain is particularly high shortly after a damaging event such as a stroke: If the functioning of one area of the brain is impaired, the adjacent areas try to take over the function of the impaired area of the brain. This is possible because the nerve cells in the brain have the ability to reconnect. In this way, the brain is able to partially compensate for damage, for example, from a stroke [27]. This competence is called neuroplasticity, also known as brain plasticity or neuronal plasticity. Thus, in order to improve outcomes, stroke patients should receive initial therapies in acute hospitalization as soon as possible. As a result of treatment with taVNS, these new connections can be enabled. Vagus nerve stimulation for stroke rehabilitation is thus related to neural substrates and their neuromodulatory effects.

Due to the mechanism of neuromodulatory reinforcement in the potentiation of the vagus nerve close to the network synapses, reinforcement learning takes place, marking network activity as significant. Neuromodulatory reinforcement is unique to recently activated synapses within the active network due to this timing, and it has no effect on inactive synapses within or outside of the network. Stimulation (VNS) thus promotes plasticity. When neuromodulators are released from neurons that terminate, this is proven to be more effective. The plasticity of the individual brain areas probably varies. A promotion of postlesional neuroplasticity by stimulating the VN is assumed [25,28].

**Figure 2:**
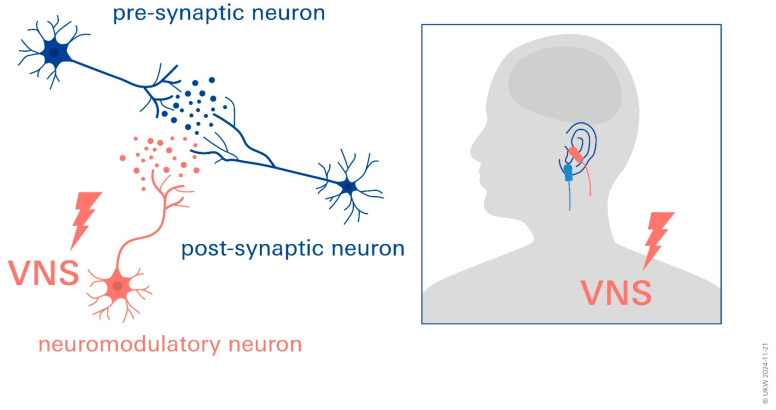
Vagus nerve stimulation for stroke rehabilitation: neuromodulatory effects promoting neuroplasticity. Image displays application of tVNS and modulation of neurotransmitter release at synapse. Red = active left tragus stimulation by taVNS device; blue = sham control stimulation sites.

taVNS in particular is associated with changes in brain structure and function that can increase the interconnectedness and effectiveness of neuronal networks [29] (Figure 2).

## 5. taVNS for Treatment of Cardiac Arrhythmias

Each year, over 12 million people worldwide suffer from stroke, which is a serious and debilitating illness. AF is a major risk factor for ischemic stroke (IS). The most prevalent chronic cardiac arrhythmia, AF, affects 2–3% of people in the USA and Europe. Compared to IS that is not related to AF, AF-related IS is linked to higher mortality, worse functional outcomes, and higher recurrence rates. Classifying the etiology of the stroke using the TOAST classification is a crucial step in lowering future stroke risk because the cause of IS influences the management decision. Cardioembolism is one of the main pathophysiologic causes of IS and frequently results from AF.

In clinical practice, arrhythmias like AF are the most prevalent. AF is a serious healthcare issue since it frequently results in heart failure or stroke. AF accounts for approximately one-third of hospitalizations caused by arrhythmias of the heart. AF has been associated with significant morbidity and a decline in quality of life [30]. Notably, in line with the aging population and the obesity epidemic, the prevalence of AF is predicted to triple over the next ten years [31,32].

Specifically, AF enhances the chance of both cardioembolic strokes and thrombosis [33]. AF may elevate the risk of an ischemic stroke due to the creation of a thrombus in the left atrium [34]. The risk of an ischemic stroke is roughly six times higher in patients with AF than in those without it, and it varies significantly based on the existence of concomitant CVD. One of the main risk factors for ischemic stroke and AF, besides aging, is obesity [35]. It is still unknown, though, how obesity, AF, and ischemic stroke are related. There is currently evidence that a significant risk factor for AF is left atrial enlargement, which is highly influenced by body weight. Through its effects on myocardial structure, autonomic function, endothelial dysfunction, and inflammatory and pro-thrombogenic states, it raises the risk of stroke associated with AF [35].

As a non-invasive supplemental treatment option, taVNS may be beneficial for some AF patients, according to a recent study [30]. The authors report that in patients with AF, taVNS significantly reduces the burden of P-wave alternans PWA and AF [30]. Furthermore, it has been demonstrated that taVNS lowers pro-inflammatory cytokines linked to AF [36,37,38,39].

## 6. Inflammation

**Figure 3:**
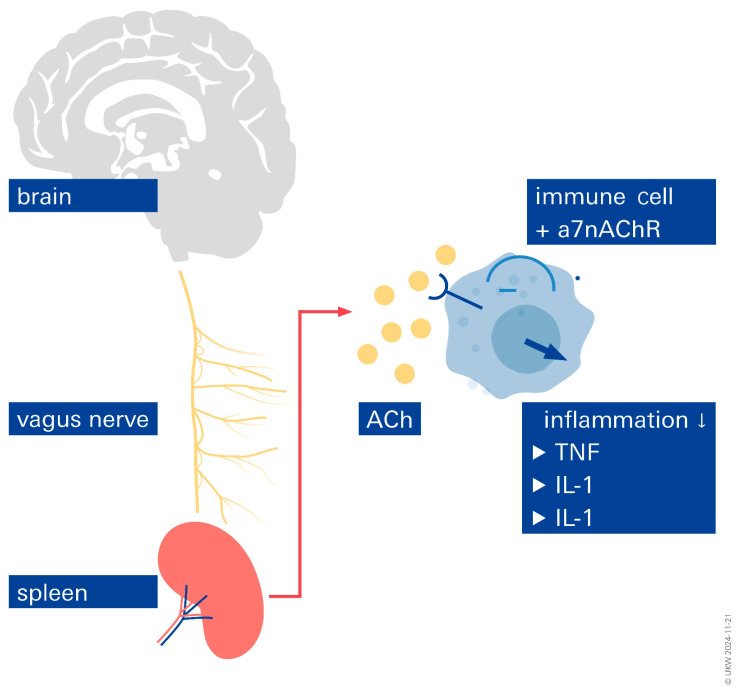
Inflammatory responses can be influenced by stimulation of the vagus nerve.

**Figure 4:**
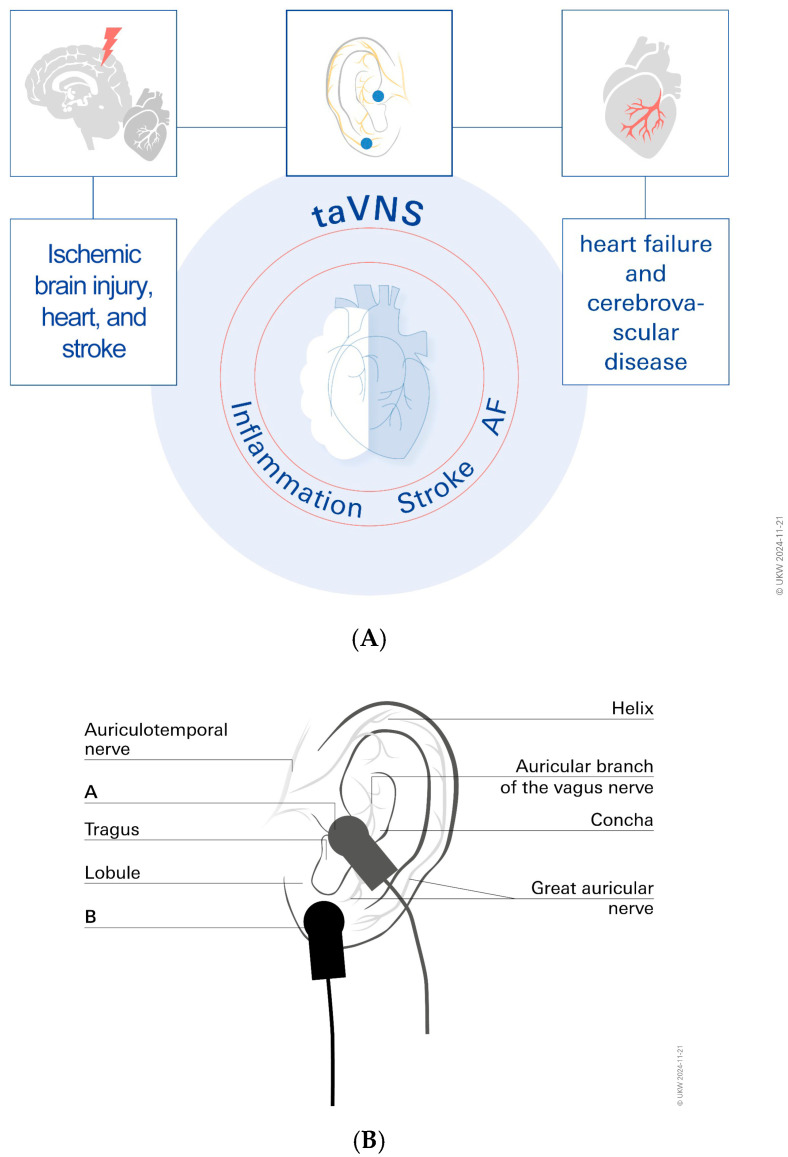
As a tool for reducing stroke infarct sizes and for beating AF, taVNS has shown promising results. (**A**). Autonomic nervous system (ANS) imbalance plays a significant role in the development of ischemic brain injury and stroke. taVNS has been shown to be effective in dampening underlying inflammatory processes. AF = atrial fibrillation; taVNS = transcutaneous auricular vagus nerve stimulation. (**B**). The cutaneous distribution of vagal fibers at the external ear (auricular branch of the vagus nerve), as described in part B, is the basis for non-invasive taVNS delivery systems. The best anatomical sites for active left tragus stimulation by the taVNS device are represented by red circles in the main image and clamps in the insert, respectively. Blue circles and clamps, on the other hand, represent sham control stimulation sites.

Inflammation appears to play a significant role in AF and stroke-related neurological disorders. The VN uses neuroendocrine and neuroimmunological pathways to reflexively reduce inflammation. This theory is supported by the observation that humoral and neuronal reflex pathways control the inflammatory process [40,41]. In general, the VN is associated with three reflex pathways that show unquestionable advantages in decreasing inflammation: the anti-inflammatory vago-vagal reflex and the cholinergic anti-inflammatory pathway have been well described [42,43]. When an infection triggers the vagus nerve fibers, acetylcholine is released at the synapses of efferent fibers. Acetylcholine can bind to the surface of macrophages and prevent the release of cytokines [44]. The anti-inflammatory function is mediated by the hypothalamic–pituitary–adrenal axis. Pro-inflammatory cytokines have been shown to be activated by infections and injuries in order to communicate with the nucleus tractus solitarius. Activation of the somatotopic area of the nucleus tractus solitarius causes an excitation cascade involving the hypothalamus and hypophysis. Finally, glucocorticoids are released by the adrenal glands. The action of glucocorticoids is a major factor in the reduction in peripheral inflammation. The anti-inflammatory splenic sympathetic pathway comes in third. Here, too, the ends of adrenergic sympathetic nerves release norepinephrine into the spleen, and efferent VN fibers are essential for this process [44]. Norepinephrine plays a crucial role in lymphocytes releasing acetylcholine (Figure 3). As was already established, acetylcholine is essential for suppressing macrophages. It can be presumed that by decreasing inflammatory responses with respect to these reflex pathways, the activity of the VN contributes significantly to maintaining homeostasis. This poses the question of whether VNS can be helpful in treating inflammatory chronic diseases and how it can maintain homeostasis and prevent immunosuppression. Regarding the anti-inflammatory effects that have been reported, numerous studies have unequivocally demonstrated that taVNS may help maintain homeostasis at the brain–heart axis by reducing pro-inflammatory markers (Figure 4). This explains why VNS therapy for stroke and atrial fibrillation should be used more widely. In fact, a recent study involving AF and stroke patients presented tVNS as a novel therapeutic option, and it was shown to lessen inflammatory reactions and improve outcomes, respectively [21,45]. Tinnitus, dementia, gastroenterology, cardiovascular disease, microvascular disease, stroke, cancer, heart failure, atrial fibrillation, and inflammation are among the new areas where taVNS shows promise.

In lymphocytes, acetylcholine is produced, and it interacts with a7nAChR (α-7 nicotinic acetylcholine receptor), which is expressed on macrophages. Through intracellular signaling, nuclear factor beta is inhibited, resulting in the suppression of cytokine production.

Recommend further readings. In order to showcase more creative projects with significant translational potential, the readings listed in the following section may be of particular interest to translational scholars and practitioners. As a treatment option for adjuvant cancer and heart failure therapy, taVNS was thoroughly examined, shedding light on a variety of delivery modes and treatment options [46]. As a direct extension of these findings, Frank et al. reported on Takotsubo syndrome and the potential role of psychosocial stress and inflammation [47]. They showed that taVNS lowers chronic pro-inflammatory markers and has a favorable effect on parasympathetic activity [47]. According to the authors, taVNS therapy offers significant potential in managing and preventing CVD, including stress-induced cardiomyopathies and specific forms of HFpEF triggered by inflammation, as well as the cardiotoxic effects of chemotherapy used in the treatment of cancer.

In the field of gastroenterology, insufficient medical treatments for gastrointestinal (GI) motility disorders and inflammatory bowel disease, coupled with their potential side effects, necessitate novel therapeutic approaches. The review by Song et al. [48] examines the potential benefits of magnetic neuromodulation, acupuncture, and VNS in the treatment of these difficult disorders. Patients who are not completely eased by conventional drugs may find a potential answer in taVNS, which provides a tailored modulation of GI motility and inflammation. In articles such as that by Paplou et al. [49], conditions such as tinnitus and age-related vestibular loss are outlined as potential candidates for taVNS in the future. The modulation of inflammation in treatment-resistant depression patients using taVNS is described in another innovative pilot study [50].

## 7. Conclusions

Previous research has confirmed the safety and efficacy of taVNS; however, a standardized protocol for treating patients with taVNS has not yet been developed, and the majority of clinical studies have small sample sizes and lack multicenter and multidisciplinary collaboration. Some of the gaps in the field’s development have also gradually come to light, owing to the quickening comprehension of the application of taVNS to human health and disease.

At the moment, taVNS is used to treat autoimmune diseases, CVD, mental diseases, and neurological diseases at the brain–heart axis. Future studies in this area are anticipated to concentrate on the use of taVNS in disorders of the central nervous system and the investigation of associated mechanisms. At the same time, the development of non-invasive neuromodulation technology presents a significant opportunity for the application of taVNS in other diseases.

## Data Availability

Not applicable.

## Abbreviations

ANS: autonomous nervous system; ABNV: auricular branch of the nervus vagus; AF: atrial fibrillation; CVD: cardiovascular disease; GI: gastrointestinal; HFpEF: heart failure preserved ejection fraction; BBB: blood–brain barrier; iVNS: invasive vagus nerve stimulation; taVNS: transcutaneous auricular vagus nerve stimulation; tVNS: transcutaneous vagus nerve stimulation; VNS: vagus nerve stimulation.
